# BCL11A Expression in Breast Cancer

**DOI:** 10.3390/cimb45040175

**Published:** 2023-03-23

**Authors:** Ewa Kątnik, Agnieszka Gomułkiewicz, Aleksandra Piotrowska, Jędrzej Grzegrzółka, Agnieszka Rusak, Alicja Kmiecik, Katarzyna Ratajczak-Wielgomas, Piotr Dzięgiel

**Affiliations:** Division of Histology and Embryology, Department of Human Morphology and Embryology, Wroclaw Medical University, 50-368 Wroclaw, Poland; ewa.katnik@student.umw.edu.pl (E.K.);

**Keywords:** BCL11A, breast cancer, BC, triple-negative breast cancer, TN, cancer cell

## Abstract

B-cell leukemia/lymphoma 11A (BCL11A) is a transcription factor that regulates the expression of genes involved in cell division or apoptosis. A link between high BCL11A expression and a worse prognosis has been demonstrated in patients with various cancers. The aim of this study was to investigate the expression pattern of BCL11A in breast cancer (BC) cases and mastopathy samples and to correlate the results with the clinicopathological data. The expression of the BCL11A protein was investigated using immunohistochemistry (IHC) on 200 cases of BC and 13 mastopathy samples. The level of BCL11A mRNA was determined using real-time PCR in 22 cases of BC and 6 mastopathy samples. The expression of BCL11A was also examined at the protein and mRNA levels in BC cell lines. A higher expression level of BCL11A in BC cases was shown compared to mastopathy samples. The expression level of BCL11A in BC cases and in the studied cell lines decreased with the increasing grade of histological malignancy (G). It was also negatively correlated with the primary tumor size. A significantly lower expression of BCL11A was found in BC that did not express estrogen or progesterone receptors and in triple-negative cases. The results of our research suggest that BCL11A may be relevant in the development of BC.

## 1. Introduction

Breast cancer (BC) is the most prevalent cancer in women worldwide. In 2020, 2.3 million new cases were diagnosed, which accounted for 24.5% of all cancers. It is also the leading cause of cancer-related mortality, with more than 600,000 deaths annually [1]. 

The main risk factors for BC are gender and age. Approximately 80% of cases occur in women over 50 years of age [1]. Risk factors also include genetic mutations, such as *TP53, CHEK, PTEN, BRCA1,* and *BRCA2*, which are detected in approximately 10% of patients. Other risk factors include early age of menarche, stimulants, the use of hormonal contraceptives, or obesity [2,3,4]. 

BC shows great diversity. Currently, there are several molecular subtypes, including luminal A, luminal B, HER2+, normal breast-like, basal-like breast cancer (BLBC), and triple-negative (TN) BC [5,6,7,8,9]. TN is characterized by the worst prognosis due to the rapid recurrence of the disease, metastasis, and the lack of specific treatment. Between 50–80% of TN cases have a basal-like phenotype, while most of the remaining cases are luminal androgen receptor (LAR) subtypes characterized by high expression of the androgen receptor (AR) gene and luminal cytokeratins, including 8 and 18 [7,8,9,10,11].

Due to the large diversity of BC, different treatments are currently available depending mainly on the subtype of cancer, the age of patients, the expression of specific genes, the presence of lymph node metastases, cancer stage, general condition, or the presence of other risk factors [12]. Targeted therapy requires adaptation of the treatment to specific gene mutations in a given patient [7,9,12]. However, despite many treatment modalities, BC is still the leading cause of cancer-related mortality in women worldwide. Therefore, it is necessary to search for new biomarkers that will facilitate the determination of the cancer subtype and allow the selection of an effective method of therapy, thus increasing the chances of successful treatment.

The *BCL11A* gene (B-cell leukemia/lymphoma 11A) encodes a transcription factor containing C2H2 zinc finger domains. It was first described by Nakamura et al. as a site for the integration of retroviruses into the genome (*Evi9*) in *BXH-2* mice, which was associated with the development of myeloid leukemia [13]. In humans, an association was shown between the translocation of the *BCL11A* locus (2p16.1) and the development of chronic B-cell lymphocytic leukemia and non-Hodgkin’s lymphoma [14,15,16,17]. The BCL11A protein has five isoforms that differ in terms of amino acid chain length, molecular weight, location in the cell, and probably function [14,18,19]. High expression of BCL11A was found in lymphatic organs, the brain, and the epidermis [15,20,21]. The participation of this protein was also confirmed in the development of lymphocytes, plasmacytoid dendritic cells, differentiation of hematopoietic stem cells, and the transition from fetal to adult hemoglobin [22,23,24,25,26]. High expression of BCL11A was also demonstrated in cancer cells, such as lymphomas, B-cell leukemias, prostate cancer, and colorectal cancer. High expression of this protein in cancer cells is associated with a worse prognosis [16,27,28,29,30,31]. In addition, BCL11A participates in the regulation of transcription of genes involved in cell division processes and apoptosis, such as *BCL2, BCL-XL, MDM2,* and *MDM4*, acting as an antiapoptotic factor [24,32,33]. Gao et al. also reported that silencing the expression of the *BCL11A* gene using siRNA reduced the viability of diffuse B-cell lymphoma (SU-DHL-6) and Burkitt lymphoma (EB1) cell lines and directed them to apoptosis [34]. Their results indicate the importance of BCL11A in carcinogenesis and suggest that this protein may play the role of an oncogene.

BCL11A is also described as a potential marker of BC. However, its role in cancer development or metastasis requires further research. To date, only a few papers have been published on the significance of BCL11A in this type of cancer. Ke et al. found a significantly higher expression of this protein in BC cells compared to normal cells. [35]. Four other studies showed overexpression of BCL11A at mRNA and protein levels in BLBC and TN subtypes [4,36,37] or only TN subtypes [11]. This was probably the result of gene amplification, hypomethylation, or the BCL11A copy number aberration [4,37]. In vitro and in vivo studies showed that silencing of BCL11A inhibited the tumorigenic potential of TN cell lines and caused tumor regression [5]. In turn, high expression of BCL11A promoted tumor formation and correlated positively with the grade of histological malignancy, clinical stage [4,35,37] and Ki-67 [11]. Patients had a worse prognosis. Metastases and relapses were more prevalent [4,37]. In addition, BCL11A is most likely crucial for the proper differentiation of BC stem cells [5]. Moody et al. proved that BCL11A interacted with histone-modifying and chromatin remodeling complexes (PRC2, NuRD, SIN3A), affecting the transcription of genes important for the development of BC stem cells (BCSCs) [38]. Silencing of *BCL11A* gene expression by miR-137 or disruption of BCL11A-DNMT1 (DNA methyltransferase 1) interaction reduced the number of cancer stem cells and inhibited cancer development [7]. Chen et al. found that the expression of the *BCL11A* gene in TN could also be regulated by non-coding circular RNA derived from epithelial stromal interaction 1 (circEPST1), which has an effect on cell proliferation and apoptosis [37]. 

The aim of this study was to investigate the location and the level of expression of BCL11A in BC cases and mastopathy samples and to correlate the results with the clinicopathological data of patients. Another aim was to determine the expression of BCL11A in BC cell lines.

## 2. Materials and Methods

### 2.1. Patient Cohort

The tissue material was obtained from patients with BC who underwent surgery in the Provincial Specialist Hospital in Wroclaw between 2004 and 2005. Archival material (paraffin blocks), including 200 cases of invasive ductal breast carcinoma and 13 mastopathy samples (control) were used for immunohistochemistry. Table 1 shows the complete clinicopathological data of patients. 

Verification of histopathological diagnosis and the grade of histological malignancy was carried out by two independent histopathologists in accordance with the WHO criteria [39]. In addition, 22 samples of invasive ductal breast carcinoma and 6 mastopathy samples fixed in RNAlater (Thermo Fisher Scientific, Waltham, MA, USA) were used to perform molecular tests. Patient clinicopathological data are given in Table 2. 

The whole study was conducted following the approval of the Bioethics Committee at the Wroclaw Medical University (KB No. 318/2018, 28 May 2018).

### 2.2. Cell Cultures

BCL11A expression was analyzed in four BC cell lines (MCF-7, SK-BR-3, MDA-MB-231, MDA-MB-468) and the MCF-10A cell line derived from mastopathy samples. All cell lines were obtained from the American Type Culture Collection (Manassas, VA, USA). MCF-10A and MCF-7 lines were cultured using the Minimum Essential Medium Eagle (MEM) Alpha Modifications (αMEM, Sigma-Aldrich, Saint Louis, MO, USA), the MDA-MB-468 line was grown in Leibovitz’s L-15 Medium (Sigma-Aldrich, Saint Louis, MO, USA), and MDA-MB-231 and SK-BR-3 were cultured in Minimum Essential Medium Eagle (EMEM, Lonza, Basel, Switzerland). All culture media were supplemented with 10% fetal bovine serum, L-glutamine, and a 1% mixture of antibiotics (penicillin G and streptomycin) (Sigma-Aldrich, Saint Louis, MO, USA). The cell lines were grown in Heracell 150i (Thermo Scientific, Waltham, MA, USA) under constant conditions (temp. 37 °C, 5% CO_2_, 95% humidity). The BC cell lines corresponded to different grades of malignancy: G1—MCF-7, G2—SK-BR-3, and G3—MDA-MB-231 [40]. The MDA-MB-468 cell line was a triple-negative (TN) breast cancer line.

### 2.3. Tissue Microarrays (TMAs)

The breast cancer TMA was constructed with archival formalin-fixed, paraffin-embedded breast tissue samples. All tumors were reviewed by two pathologist-researchers (Piotr Dzięgiel, Aleksandra Piotrowska). In the first stage, specimens of 4 μm thickness were made. They were stained with hematoxylin and eosin. Then, the specimens were scanned using the Pannoramic Midi II histological scanner (3DHistech, Budapest, Hungary), and for each case, 3 representative spots with a diameter of 1.5 mm were selected. From the selected spots, tissue microarrays were made using TMA Grand Master equipment (3D Histech, Budapest, Hungary).

### 2.4. Immunohistochemistry (IHC)

Paraffin blocks of tissue microarrays were cut into sections of 4 μm thickness. They were deparaffinized and hydrated, and epitopes were exposed by boiling at 97 °C for 20 min in the high-pH Target Retrieval Solution in Dako PT Link (Dako, Glostrup, Denmark). Endogenous peroxidase was blocked by incubating sections in EnVisionTM FLEX Peroxidase-Blocking Reagent (Dako, Glostrup, Denmark) for 5 min at room temperature. Specific primary mouse anti-BCL11A monoclonal antibodies (ab19489, Abcam, Cambridge, UK) diluted 1:200 in FLEX Antibody Diluent (Dako, Glostrup, Denmark) were used to detect BCL11A expression. The sections were incubated for 20 min at room temperature. After rinsing in the EnVision FLEX Wash Buffer (Dako, Glostrup, Denmark), the secondary antibody conjugated with EnVisionTM FLEX/horseradish peroxidase (HRP; Dako, Glostrup, Denmark) was applied for 20 min at room temperature. The sections were incubated for 10 min at room temperature with a substrate for peroxidase 3,3′-diaminobenzidine (DAB) to obtain the color reaction. The preparations were stained with hematoxylin (EnVisionTM FLEX Hematoxylin; Dako, Glostrup, Denmark) for 7 min at room temperature. Finally, they were dehydrated in ethanol solutions with increasing concentrations (70%, 96%, 99.9%) and xylene. IHC reactions were performed using Dako Autostainer Link48 (Dako, Glostrup, Denmark). The negative control was prepared without the use of the primary antibody.

### 2.5. Evaluation of Immunohistochemical Reactions

The expression level of BCL11A was analyzed by two independent investigators (Piotr Dzięgiel and Ewa Kątnik) using the BX-41 light microscope (Olympus, Tokyo, Japan). The evaluation of IHC staining was performed at ×200 magnification. For each case, 3 representative spots (1.5 mm diameter) were evaluated. Each spot contained an average of 4000–6000 cells. The intensity of the cytoplasmic reaction was determined using the 12-point semi-quantitative immunoreactive score (IRS) according to Remmele and Stegner (0–12 points) [41]. It is the product of two variables: the percentage of positive cells and the intensity of the color reaction (A × B, Table 3). 

A 3-point scale was used to assess the nuclear reaction taking into account the percentage of positive cells: 0 points—≤5%, 1 point—6–25%, 2 points—26–50%, 3 points—>50% of positive cells [42].

### 2.6. RNA Isolation and cDNA Synthesis

Total RNA was isolated from BC cases, mastopathy samples and cell line deposits (MCF-7, SK-BR-3, MDA-MB-231, MDA-MB-468, MCF-10A) using RNeasy Mini Kit reagents (Qiagen, Hilden, Germany) according to the manufacturer’s instructions. The NanoDrop1000 spectrophotometer (ThermoFisher, Waltham, MA, USA) was used to measure the concentration and purity of RNA. Reverse transcription reactions were performed using the High-Capacity cDNA Reverse Transcription Kit (Applied Biosystems, Foster City, CA, USA) in the SimpliAmpTM Thermal Cycler (Applied Biosystems, Foster City, CA, USA). The conditions were as follows: 10 min at 25 °C, 120 min at 37 °C, and 5 min at 85 °C.

### 2.7. Real-Time PCR

The levels of BCL11A mRNA in BC cases, mastopathy samples, and the selected cell lines (MCF-7, SK-BR-3, MDA-MB-231, MDA-MB-468, MCF-10A) were assessed using real-time PCR (7500 Real-Time PCR System; Applied Biosystems, Foster City, CA, USA). β-actin was the reference gene that was used to normalize the results. The TaqMan Gene Expression Master Mix (Applied Biosystems, Foster City, CA, USA) and the sets of specific primers and TaqMan probes were used for BCL11A (Hs00256254_m1, Hs01093196_m1, Hs00250581_s1) and for β-actin (Hs99999903_m1) (Applied Biosystems, Foster City, CA, USA). The reactions were carried out in triplicate under the following conditions: polymerase activation at 50 °C for 2 min; initial denaturation at 95 °C for 10 min, followed by 45 cycles involving denaturation at 95 °C for 15 s; and primer attachment and DNA synthesis at 60 °C for 1 min. The ΔΔCt method was used to calculate the relative mRNA expression level of BCL11A.

### 2.8. Immunofluorescence (IF)

Immunofluorescence was used to assess the expression level and the location of BCL11A in cell lines (MCF-10A, MCF-7, SK-BR-3, MDA-MB-231, MDA-MB-468). Initially, 2 × 104 cells were seeded into each well of the Millicell EZ 8-well glass slide (Merck, Darmstadt, Germany; PEZGS0816). After 48 h of culture under standard conditions, the cells were fixed with an acetone–methanol mixture (1:1) at 4 °C for 15 min. The cells were incubated in 3% BSA in 0.4% TBST buffer for 45 min at room temperature to block nonspecific binding sites. Specific primary mouse anti-BCL11A monoclonal antibodies (ab19489, Abcam, Cambridge, UK; 1:200) were used (overnight incubation at 4 °C). The negative control was incubated only from PBS. The next step was the application of secondary antibodies (Alexa Fluor 488; ab150113, Abcam, Cambridge, UK; 1:4000) for 1 h at room temperature. Finally, the specimens were enclosed in Fluoroshield Mounting Medium with DAPI (Abcam, Cambridge, UK; ab104139). Evaluation of BCL11A expression in cell lines was performed using Confocal Laser Scanning Microscope Fluoview FV3000 coupled with CellSense software (Olympus, Hamburg, Germany).

### 2.9. Statistical Analysis

The results were analyzed using Statistica 13 (StatSoft, Krakow, Poland) and Prism 5.0 (GraphPad Software, San Diego, CA, USA). The Shapiro–Wilk test was used to check whether the data presented a normal distribution. The non-parametric Mann–Whitney U test was used to compare data in the groups. The Spearman’s rank correlation was used to examine the relationship between BCL11A expression and the selected factors. The Tukey’s Multiple Comparison Test was used to compare fluorescence intensity in cell lines. The Kaplan–Meier method was used to perform survival analysis and the significance of differences was determined using the log-rank test. The cut-off point for BCL11A expression was determined based on the median (0–3 vs. 4–12 pts). Results of *p* < 0.05 were considered statistically significant.

## 3. Results

### 3.1. Expression of BCL11A in Breast Cancer Cells and Mastopathy Samples

**Figure 1:**
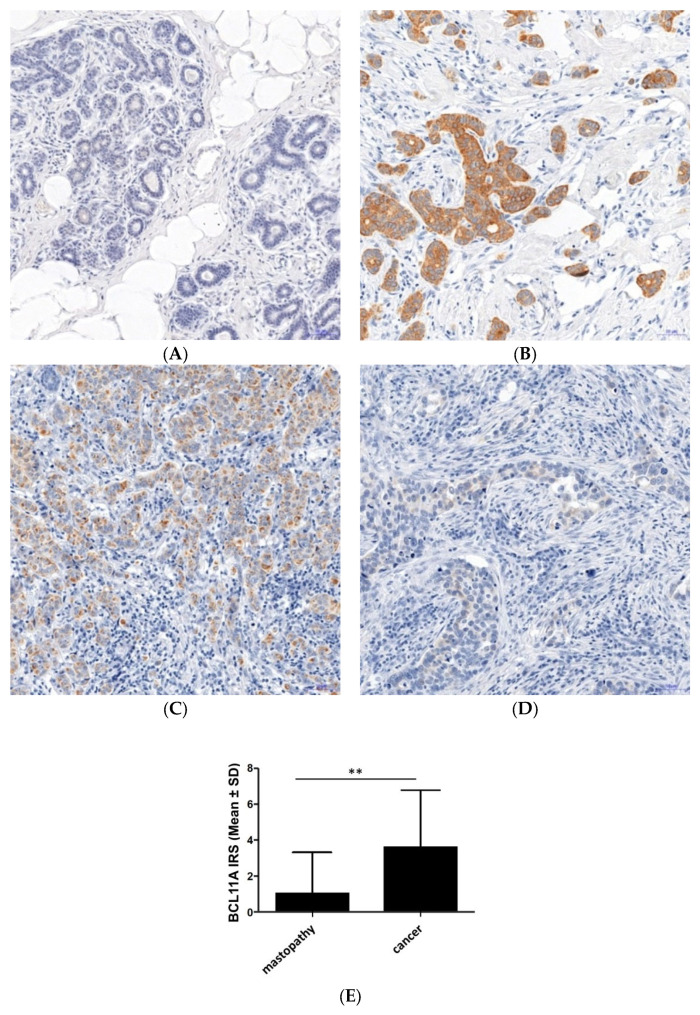
Expression of B-cell leukemia/lymphoma 11A (BCL11A) in (**A**) mastopathy samples and (**B**–**D**) breast cancer cases using immunohistochemistry. The expression level of BCL11A decreased with an increasing grade of histological malignancy (G) of breast cancer cases. It was high in (**B**) G1, and it was gradually lower in (**C**) G2 and (**D**) G3. (**A**) Very low expression levels were found in mastopathy samples. Magnification ×200. (**E**) Comparison of BCL11A expression in mastopathy samples and breast cancer samples (** *p* = 0.001); Mann–Whitney U test.

Immunohistochemical reactions showed cytoplasmic expression of BCL11A in 158 cases of BC (79%) (Figure 1B–D).

The mean expression was 3.655 ± 3.124. The obtained values were divided into two groups: 0–3 points—low expression of BCL11A (107) and 4–12 points—high expression of BCL11A (93). In addition, nuclear expression of BCL11A was demonstrated in 7 BC cases. A similar location of this protein was found in lymphocytes, which were inflammatory infiltrates. A positive cytoplasmic immunohistochemical reaction was found in 30.77% of mastopathy samples. The remaining cases (69.23%) did not express this protein (Figure 1A). Statistical analyses showed significantly higher expression of BCL11A in BC cases (3.655 ± 3.124) compared to mastopathy samples (1.077 ± 2.253, ** *p* = 0.001, Figure 1E). 

**Figure 2:**
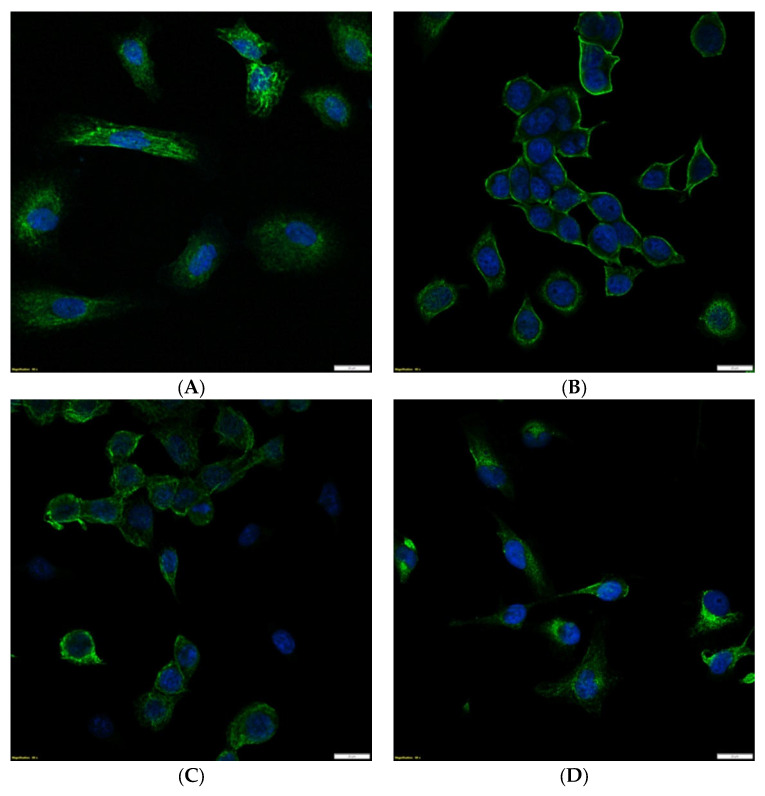

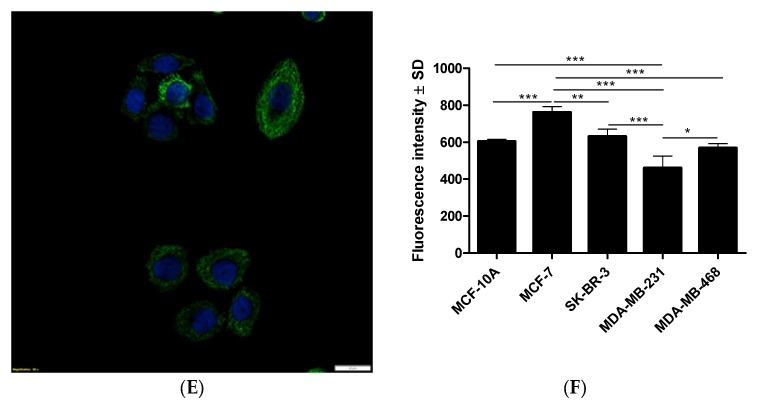
Immunofluorescence reaction detecting cytoplasmic expression of B-cell leukemia/lymphoma 11A (BCL11A) (green) in cell lines. Cell nuclei were visualized by DAPI dye (blue nuclei). (**A**) MCF-10A cell line derived from mastopathy sample. (**B**–**E**) Breast cancer cell lines. The expression level of BCL11A decreased with an increasing grade of histological malignancy (G). It was high in (**B**) MCF-7 (G1), and it was gradually lower in (**C**) SK-BR-3 (G2) and (**D**) MDA-MB-231 (G3). (**E**) MDA-MB-468 is the triple-negative breast cancer cell line. Magnification ×600. (**F**) The expression level of BCL11A in the cell lines (IF) was assessed using confocal microscopy (*** *p* < 0.0001, ** *p* < 0.01, * *p* < 0.05); Tukey’s Multiple Comparison Test.

Based on immunofluorescence analyses using confocal microscopy, cytoplasmic expression of BCL11A was demonstrated in all studied cell lines: MCF-10A, MCF-7, SK-BR-3, MDA-MB-231, and MDA-MB-468 (Figure 2A–E). The MCF-10A cell line derived from the mastopathy sample presented with significantly lower expression level of BCL11A compared to the MCF-7 cell line (603.50 ± 10.52 vs. 761.14 ± 31.82, *** *p* < 0.0001) but significantly higher expression compared to the MDA-MB-231 cell line (603.50 ± 10.52 vs. 460.75 ± 56.09, *** *p* < 0.0001). There were no statistically significant differences in BCL11A expression between the MCF-10A line and other cancer cell lines. Reactions carried out on cancer cells showed significantly higher BCL11A expression in the MCF-7 line (761.14 ± 31.82) compared to the SK-BR-3 (630.58 ± 34.75, ** *p* < 0.01), MDA-MB-468 (568.37 ± 21.14, *** *p* < 0.0001), and MDA-MB-231 (460.75 ± 56.09, *** *p* < 0.0001) lines. The comparison of the expression level of BCL11A in the cell lines is presented in Figure 2F.

### 3.2. The Relationship between BCL11A Expression and Clinicopathological Factors

**Figure 3:**
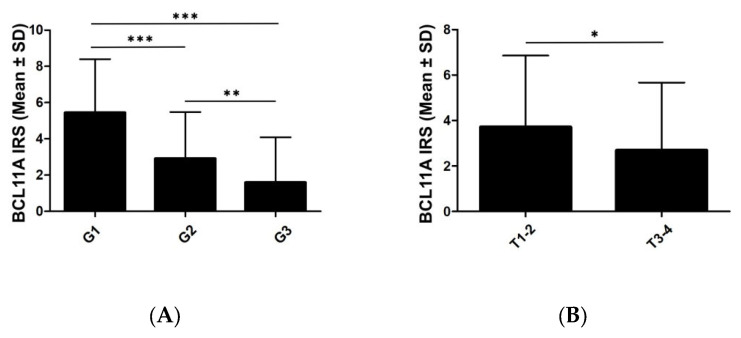

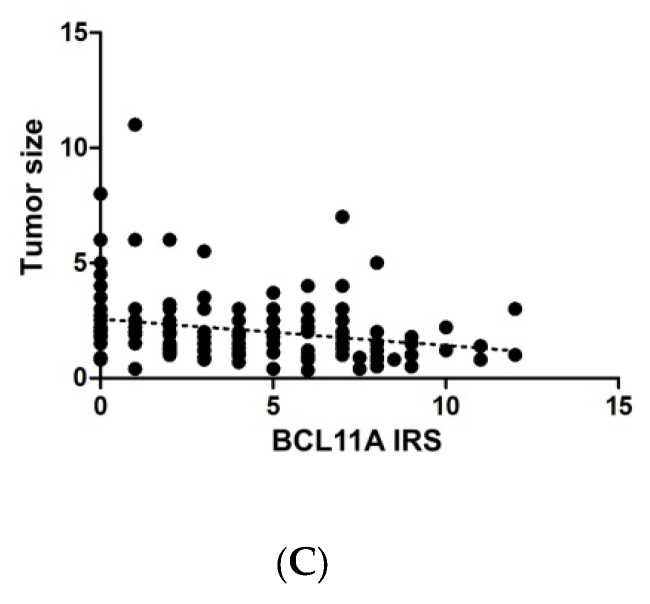
The level of cytoplasmic expression of B-cell leukemia/lymphoma 11A (BCL11A) (immunohistochemical reactions) in relation to clinicopathological factors. The expression level of BCL11A in breast cancer cases depending on (**A**) the grade of histological malignancy (G) (*** *p* < 0.0001, ** *p* = 0.0011), (**B**) the size of the primary tumor (T) (* *p* = 0.048); Mann–Whitney U test. (**C**) Correlation of BCL11A expression with the tumor size in cm (r = −0.34; *** *p* < 0.0001); Spearman rank correlation.

BCL11A expression levels were shown to decrease gradually with increasing histological malignancy grade (G) of BC. The mean BCL11A expression (IHC) was significantly higher in cases with G1 (5.432 ± 2.956) compared to G2 (3.055 ± 2.748, *** *p* < 0.0001) and G3 (1.593 ± 2.498, *** *p* < 0.0001). Statistical significance was also demonstrated between G2 and G3 (** *p* = 0.0011; Figure 1B–D and Figure 3A).

In the cell lines, the highest expression of BCL11A (IF) was found in the MCF-7 line, which corresponded to BC with G1 (761.14 ± 31.82), while lower expression was observed in the SK-BR-3 line—which corresponded to BC with G2 (630.58 ± 34.75, ** *p* < 0.01)—and in MDA-MB-231 line, corresponding to BC with G3 (460.75 ± 56.09, *** *p* < 0.0001). Statistical significance was also demonstrated between SK-BR-3 (G2) and MDA-MB-231 (G3) lines (*** *p* < 0.0001; Figure 2F).

The analysis of BCL11A expression also showed a statistically significant inverse relationship with the primary tumor size (* *p* = 0.048; Figure 3B) and the tumor size in cm (r = −0.34, *** *p* < 0.0001; Figure 3C). However, no association was found between BCL11A expression level and patient age, tumor stage, or lymph node metastasis (Table 4).

### 3.3. The Relationship between BCL11A Expression and the Presence of Receptors for Steroid Hormones and HER2

**Figure 4:**
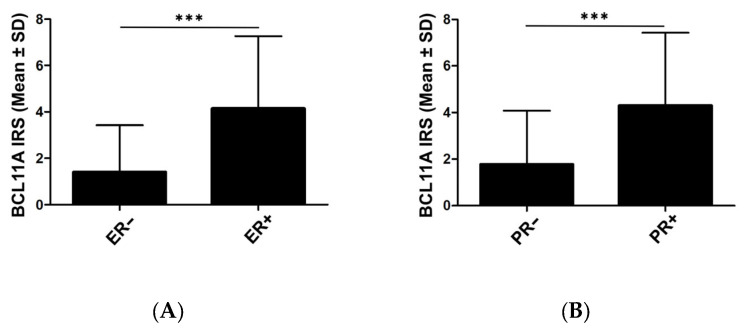

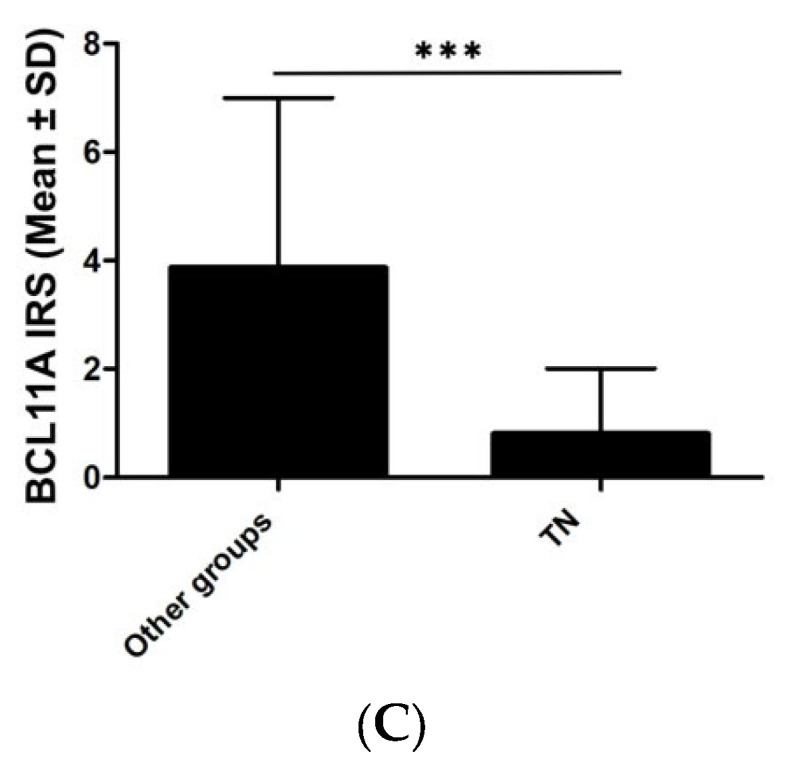
The level of cytoplasmic expression of B-cell leukemia/lymphoma 11A (BCL11A) (immunohistochemical reactions) in relation to clinicopathological factors. The expression level of BCL11A in breast cancer cases depending on (**A**) the presence of estrogen receptors (ERs) (*** *p* < 0.0001), (**B**) the presence of progesterone receptors (PRs) (*** *p* < 0.0001); Mann–Whitney U test. (**C**) Comparison of BCL11A expression in triple-negative (TN) cases and other cases of breast cancer (*** *p* < 0.0001); Mann–Whitney U test.

Significantly higher levels of BCL11A expression were found in BC cases showing the expression of estrogen receptors (ER+) compared to cases that did not show the expression of these receptors (ER−) (*** *p* < 0.0001; Figure 4A). The BCL11A expression level for ER+ cases presented the mean values (4.196 ± 3.086), while the BCL11A level for ER− cases was 1.744 ± 2.564. Similar results were obtained when comparing cases of cancers with PR+ and PR− (*** *p* < 0.0001; Figure 4B). The mean values of BCL11A for PR+ cases were 4.377 ± 3.146, and 1.904 ± 2.337 for PR−. According to the above results, significantly lower levels of the protein were shown in cases of TN breast cancers (TN, *** *p* < 0.0001, Figure 4C) compared to the other cases. However, no significant differences were found in BCL11A expression levels between the cases with HER2 expression (HER2+) and those without HER2 expression (HER2−). 

### 3.4. BCL11A mRNA Expression in Breast Cancer and Cell Lines

BCL11A mRNA expression was demonstrated in all BC and mastopathy cases. There were no statistically significant differences in BCL11A mRNA expression in cancers (RQ 74.302 ± 95.743) compared to mastopathy samples (RQ 35.730 ± 24.378, *p* = 0.737). No significant correlations were found between BCL11A mRNA expression levels and clinicopathological factors (Table 5). 

High expression of BCL11A mRNA was demonstrated in the SK-BR-3 line—which corresponded to G2 BC (RQ 144.212 ± 49.317)—and in the MDA-MB-468 line, representing TN cases (TN; RQ 64.714 ± 16.827). Lower levels of BCL11A mRNA were found in the MCF-7 (RQ 1.816 ± 1.309) and MDA-MB-231 (RQ 1.178 ± 0.522) lines, which corresponded to G1 and G3, respectively. In the MCF-10A cell line derived from mastopathy samples, BCL11A mRNA expression was RQ 25.763 ± 4.550.

### 3.5. The Relationship between BCL11A Expression and Overall Survival

**Figure 5:**
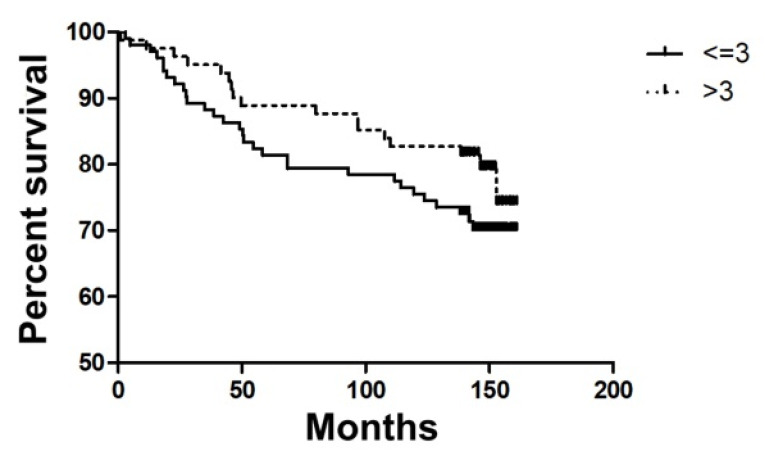
Kaplan–Meier curves showing the prognostic effect of B-cell leukemia/lymphoma 11A (BCL11A) expression level (immunohistochemical reactions) on the overall survival of patients with breast cancer (*p* = 0.1389). The analysis was performed using the log-rank test. The cutoff point for BCL11A expression was determined based on the median (0–3 vs. 4–12 points).

The log-rank analysis did not show significant relationship between the expression level of BCL11A and overall survival of patients with BC. We observed that patients with higher BCL11A expression (4–12 points) showed a slightly longer survival time compared to those with low protein levels (0–3 points). However, the differences were not statistically significant (*p* = 0.1389; Figure 5). In addition, the survival analysis was performed using an online analysis tool, the Kaplan–Meier Plotter [43]. The results obtained for both BCL11A protein (N = 65) and BCL11A mRNA (N = 2976) were not statistically significant (*p* = 0.3 and *p* = 0.29, respectively; Figure S1).

## 4. Discussion

BC is the most prevalent malignant tumor diagnosed each year in women worldwide. It is also the leading cause of cancer-related mortality [1,2]. The large diversity of molecular subtypes of this cancer is one of the reasons for the difficulties related to its treatment [6,7,8,9,10]. The selection of effective therapy requires considering a molecular subtype of cancer, including the expression of specific proteins. One of them is BCL11A. Recently, there has been research suggesting a link between high BCL11A expression and tumor resistance to tamoxifen therapy. The cause was probably the activation of the PI3K/AKT and MAPK/ERK pathways involved in tumor cell growth and metastasis [44,45]. In addition, the results of the study suggested the participation of BCL11A in the development of various types of cancer, such as lung cancer, B-cell lymphomas and leukemias, and prostate and colorectal cancer [16,27,28,29,30,31]. However, little is known about the role of BCL11A in BC.

Therefore, in our study, we examined the expression of BCL11A in BC cases and analyzed the relationship between the expression level of this protein and clinicopathological factors.

We showed significantly higher cytoplasmic expression of BCL11A in BC compared to mastopathy samples, which were used as the control. Our results are in line with the findings of Zhu et al. and Ke et al., who showed significantly higher expression of BCL11A in BC compared to controls, which were normal tissues from the tumor environment [35,46]. Both the results of our research and the observations of Zhu et al. and Ke et al. suggest that BCL11A may be relevant in carcinogenesis. This is due to the fact that this protein is probably responsible for disorders of cell proliferation, cell cycle, and apoptosis by activating the Wnt/β-catenin pathway [46] and antiapoptotic proteins, such as BCL2, BCL2-XL and MDM2 (a negative regulator of the p53 protein) and for inhibiting p21 and p53 proteins responsible for proper apoptosis and cell cycle [31]. Angius et al. also showed high expression of BCL11A mRNA; its isoforms, BCL11A-XL, BCL11A-L, and BCL11A-S; as well as the protein, but in clinical material of TN breast cancers. In this research, nuclear expression of the examined protein was observed [11]. The results are in line with our findings, as we also observed the nuclear expression of BCL11A in 7 TN cases (44%).

Other studies have shown that BCL11A can be silenced by microRNAs (such as miR-30a) in normal lung tissue [31]. Jiang et al. noticed low levels of miR-30a in non-small cell lung cancer (NSCLC). This resulted in an increase in BCL11A expression and possibly a disruption of the above processes associated with the development of lung cancer [31]. Similarly, Lulli et al. demonstrated that inhibition of miR-486-3p expression in esophageal, pancreatic, and lung cancer cells resulted in an increase in BCL11A levels and promoted tumor development [47]. In contrast, long non-coding RNA uc.57 decreased BCL11A levels in the breast cancer cells surrounding the tumor. However, as in the above cases, the concentration of uc.57 in BC is quite low and does not fulfill its function, which results in an increase in the concentration of BCL11A and a disturbance of normal processes in the cell [45]. Similarly, Zhang et al. demonstrated that inhibition of miR-574-5p expression in TNBC cells resulted in an increase in BCL11A levels and promoted proliferation, migration and EMT in TNBC cells [48]. The results are in line with Gong et al.’s findings, as they also observed inhibition of has-miR-190b expression in BC cells, which resulted in an increase in BCL11A levels and promoted tumor development [49]. These results suggest a very diverse way of regulating BCL11A expression, which requires further research that could confirm our findings.

In addition, we showed that BCL11A expression levels decreased gradually with the increase in the grade of histological malignancy of BC. Similar relationships were observed in the cell lines of this cancer. Our results indirectly confirm the findings of Moody et al., who proved that BCL11A interacted with histone-modifying and chromatin-remodeling complexes (PRC2, NuRD, SIN3A), which affected the transcription of genes important for the development of BCSCs [38]. This is crucial for the initiation of tumor transformation, hence probably high expression of this protein in BC cases with the lowest grade of malignancy (G1). The results of our study are not consistent with the findings of Angius et al. and Khaled et al. Angius et al. did not observe statistically significant correlation of BCL11A with the grade of histological malignancy of BC [11], whereas Khaled et al. presented a positive correlation of BCL11A with the grade of histological malignancy of BC [5]. These discrepancies may be caused by methodological differences, such as IHC reactions and the methods for assessing them or a different selection of the experimental material. The inverse relationship between BCL11A expression and the grade of malignancy (G) is consistent with a significant negative correlation between the expression of this protein and the tumor size that we demonstrated in our study. A similar trend was observed by Jiang et al. in NSCLC [31]. This suggests the participation of the BCL11A protein in carcinogenesis. This was confirmed by Angius et al., who showed an association between high levels of BCL11A and Ki-67 [11]. This was also confirmed by Yu et al., who demonstrated an association between high levels of BCL11A and the inhibition of apoptosis of cancer cells [24]. An important role in this process is played by the activation of BCL2 and BCL-XL, which belong to antiapoptotic proteins that inhibit the release of cytochrome c and the apoptosis-inducing factor (AIF) from the mitochondria [24]. Another mechanism is the induction of expression of MDM4 and MDM2 proteins, which function as negative regulators of the p53 protein [24]. These studies were also confirmed by the results obtained in two cell lines of lymphoma (EB1 and SU-DHL-6), which showed that silencing the expression of the *BCL11A* and *BCL2* genes by siRNA reduced the viability of cells, inhibited their growth, and directed them to apoptosis [34]. Nakamura et al., who conducted research on the normal mouse fibroblast cell line (NIH/3T3), showed that increased expression of BCL11A was associated with tumor transformation of these cells [13]. The above results may indicate that BCL11A may act as an oncogene.

In our study we did not show statistically significant relationship between the expression level of BCL11A and overall survival of patients with BC. However, we observed a trend indicating that higher expression of this protein may be associated with slightly longer survival time. The relationship between higher expression level of BCL11A and longer overall survival was observed by Angius et al. in their study of TN breast cancer cases [11]. However, different results were described by Chen et al., who observed that higher levels of BCL11A correlated with a worse prognosis, metastasis rate, and recurrence [37]. The survival analysis carried out by those authors was limited to TN cases, as in the work of Angius et al. [11]. These conflicting results make it impossible to clearly determine the relationship between BCL11A expression and overall survival of BC patients and suggest a need for further investigation. The results of our study are consistent with the findings of Hayashi et al., who showed a relationship of high expression of BCL11A and other markers (CDK2, HER2, CDKN1A) with a lower risk of bone metastasis in patients with BC [50]. In turn, analyzing cases of diffuse large-B-cell lymphoma (DLBCL), Pulford et al. observed that the lack of expression of the BCL11A-XL isoform in cancer cells correlated with shorter survival [51]. Our results are also consistent with the studies of Jiang et al. and Zhang et al. on NSCLC. High BCL11A expression correlated with longer disease-free survival and overall survival [19,31]. Additionally, patients with tumors with low expression of BCL11A-XL showed lower survival [19].

In conclusion, we have shown that BCL11A can be important in the development of BC. The negative correlations between the expression level of BCL11A, the grade of histological malignancy (G), and the size of the tumor allow us to assume that BCL11A plays a role in the initiation of tumor transformation. In addition, further studies on BCL11A in BC are warranted to better explain its mechanism of action in this condition.

## Figures and Tables

**Table 1:** Clinicopathological data of patients from whom the material was collected for immunohistochemistry.

Parameters	All Cases	
	N = 200	%
Age		
<58	110	55.0%
≥58	90	45.0%
Grade of histological malignancy (G)		
G1	73	36.5%
G2	73	36.5%
G3	43	21.5%
no data	11	5.5%
Size of the primary tumor (T)		
pT1	110	55.0%
pT2	58	29.0%
pT3	4	2.0%
pT4	20	10.0%
no data	8	4.0%
Tumor size (cm)		
<2 cm	94	47.0%
≥2 cm	98	49.0%
no data	8	4.0%
Lymph node metastases (N)		
pN0	68	34.0%
pN1	27	13.5%
pN2	17	8.5%
pN3	6	3.0%
no data	82	41.0%
Cancer stage		
I	44	22.0%
II	40	20.0%
III	37	18.5%
no data	79	39.5%
ER status		
positive	153	76.5%
negative	43	21.5%
no data	4	2.0%
PR status		
positive	134	67.0%
negative	57	28.5%
no data	9	4.5%
HER2 status		
positive	97	48.5%
negative	95	47.5%
no data	8	4.0%
Triple-negative (TN) breast cancer		
yes	16	8.0%
no	178	89.0%
no data	6	3.0%
Survival		
survivors	146	73.0%
deceased	52	26.0%
no data	2	1.0%

**Table 2:** Clinicopathological data of patients from whom the material was collected for molecular studies.

Parameters	All Cases	
	N = 22	%
Age		
<61	7	31.8%
>61	8	36.4%
no data	7	31.8%
Grade of histological malignancy (G)		
G1	5	22.7%
G2	9	40.9%
G3	8	36.4%
Size of the primary tumor (T)		
pT1	11	50.0%
pT2	10	45.5%
pT3	1	4.5%
Lymph node metastases (N)		
pN0	9	40.9%
pN1	11	50.0%
pN2	1	4.5%
pN3	1	4.5%
Cancer stage		
I	3	13.6%
II	10	45.5%
III	3	13.6%
no data	6	27.3%
ER status		
positive	14	63.6%
negative	6	27.3%
no data	2	9.1%
PR status		
positive	12	54.5%
negative	8	36.4%
no data	2	9.1%
HER2 status		
positive	10	45.5%
negative	10	45.5%
no data available	2	9.1%
Triple-negative (TN) breast cancer		
yes	4	18.2%
no	16	72.7%
no data	2	9.1%
Survival		
survivors	5	22.7%
deceased	7	31.8%
no data	10	45.5%

**Table 3:** Semi-quantitative immunoreactive score (IRS), according to Remmele and Stegner [41]. The final score is the product of factors A and B, ranging from 0 to 12.

Factor A		Factor B	
Points	Percentage of Positive Cells	Points	Reaction Intensity
0	0%		
1	≤10%	0	lack
2	11–50%	1	low
3	51–80%	2	medium
4	>80%	3	strong

**Table 4:** The relationship of B-cell leukemia/lymphoma 11A (BCL11A) protein expression with clinicopathological factors in the group of patients with breast cancer.

Parameters	All Cases (N = 200)	
	Mean Expression Value ± SD	*p*
Age		0.1685
<58	3.836 ± 3.235	
≥58	3.433 ± 2.986	
Grade of histological malignancy (G)		<0.0001
G1	5.432 ± 2.956	
G2	3.055 ± 2.748	
G3	1.593 ± 2.498	
Size of the primary tumor (T)		0.0479
pT1	4.318 ± 3.187	
pT2	2.879 ± 2.908	
pT3	1.500 ± 1.291	
pT4	2.800 ± 3.172	
Tumor size (cm)		<0.0001
<2 cm	4.511 ± 3.224	
≥2 cm	2.888 ± 2.864	
Status of lymph node metastases (N)		0.6905
pN0	3.081 ± 3.100	
pN1	4.407 ± 3.522	
pN2	2.588 ± 2.210	
pN3	2.500 ± 3.209	
Cancer stage		0.0837
I	3.784 ± 3.133	
II	3.325 ± 3.482	
III	2.378 ± 2.586	
ER status		<0.0001
positive	4.196 ± 3.086	
negative	1.744 ± 2.564	
PR status		< 0.0001
positive	4.377 ± 3.146	
negative	1.904 ± 2.337	
HER2 status		0.8222
positive	3.500 ± 3.230	
negative	3.657 ± 3.120	
Triple-negative (TN) breast cancer		<0.0001
yes	1.000 ± 1.414	
no	3.921 ± 3.153	

**Table 5:** The relationship between B-cell leukemia/lymphoma 11A (BCL11A) mRNA expression with clinicopathological factors in the group of patients with breast cancer.

Parameters	All Cases (N = 22)	
	RQ ± SD	*p*
Age		0.4587
<61	41.591 ± 3.459	
>61	9.506 ± 7.516	
Grade of histological malignancy (G)		0.4339
G1	17.258 ± 5.559	
G2	50.933 ± 3.855	
G3	11.561 ± 8.356	
Size of the primary tumor (T)		0.9955
pT1	21.232 ± 5.821	
pT2-pT3	25.351 ± 6.761	
Lymph node metastases (N)		0.3899
pN0	34.514 ± 5.117	
pN1-pN3	17.660 ± 6.792	
Cancer stage		0.2427
I	32.810 ± 4.581	
II-III	20.045 ± 6.902	
ER status		0.2313
positive	14.028 ± 6.577	
negative	54.702 ± 4.375	
PR status		0.2189
positive	11.830 ± 6.339	
negative	50.350 ± 4.786	
HER2 status		0.1839
positive	10.593 ± 6.808	
negative	42.073 ± 4.808	
Triple-negative (TN) breast cancer		0.0890
yes	78.886 ± 2.917	
no	15.205 ± 6.516	

## Data Availability

The raw data and the analytic methods will be made available to other researchers for purposes of reproducing the results in their own laboratories upon reasonable request. To access protocols or datasets, contact agnieszka.gomulkiewicz@umw.edu.pl.

## Supplementary Materials

The following supporting information can be downloaded at: https://www.mdpi.com/article/10.3390/cimb45040175/s1, Figure S1. Kaplan–Meier curves showing the prognostic effect of BCL11A protein expression (A) and mRNA level (RNA-Seq) (B) on the overall survival of patients with breast cancer. The analysis was performed using the Kaplan–Meier Plotter.
