# Supplementary material for: BCL11A Expression in Breast Cancer
Source: Curr Issues Mol Biol. 2023 Mar 23;45(4):2681–98. doi: 10.3390/cimb45040175 (PMC10137054; doi:10.3390/cimb45040175)
Supplement: Supplementary file 1 [file cimb-45-00175-s001.zip › cimb-2257348-supplementary.pdf]

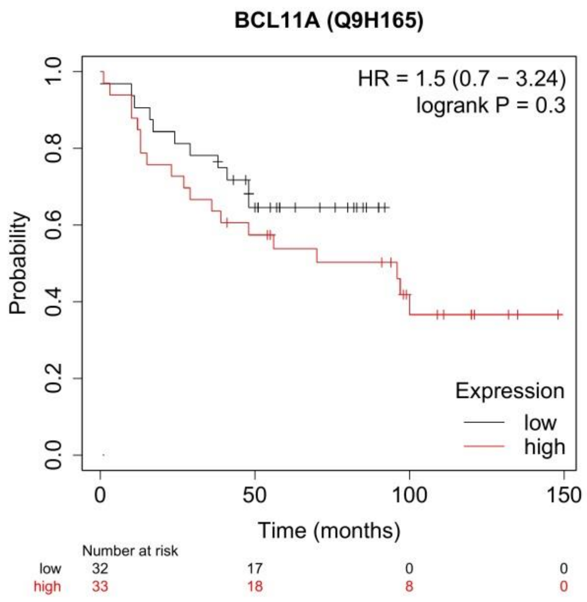

A

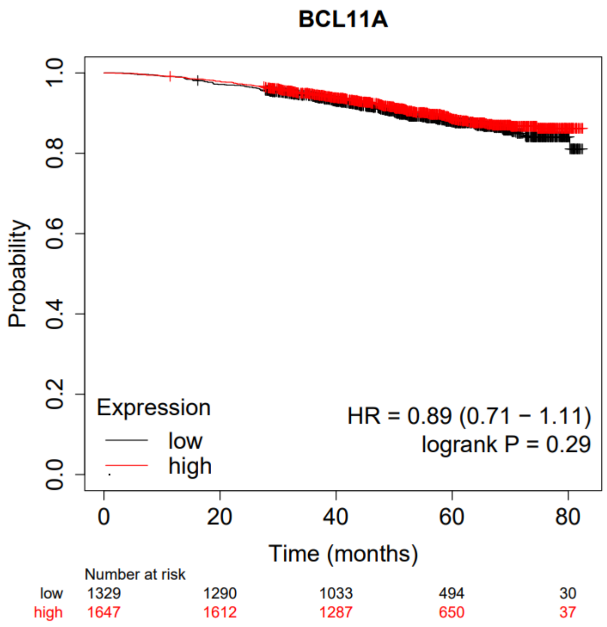

B

Figure S1. Kaplan-Meier curves showing the prognostic effect of B-cell leukemia/lymphoma 11A (BCL11A) protein expression (**A**) and mRNA level (RNA-Seq) (**B**) on the overall survival of patients with breast cancer. The analysis was performed using the Kaplan-Meier Plotter.
